# Factors associated with unmet medical needs among people with disabilities using Andersen's model in South Korea

**DOI:** 10.3389/fpubh.2026.1871910

**Published:** 2026-08-12

**Authors:** Ye-Soon Kim, Mi Jin Kim, Seon-Deok Eun

**Affiliations:** Department of Healthcare and Public Health Research, National Rehabilitation Center, Seoul, Republic of Korea

**Keywords:** Andersen's model, enabling factors, people with disabilities, predisposing factor, unmet medical needs

## Abstract

**Objective:**

This study applied Andersen's behavioral model to identify unmet medical needs and related associated factors among people with disabilities.

**Methods:**

The analysis was conducted on 7,581 people with disabilities aged ≥20 years, obtained from the 2023 Survey on People with Disabilities in Korea.

**Results:**

The results showed that 17.2% of people with disabilities reported wanting to visit a medical institution but were unable to do so. The main reasons for unmet medical needs were “difficulty in transportation”, “economic reasons”, and “lack of time”, which accounted for 73.0% of cases. According to Andersen's model, the predisposing factors associated with unmet medical needs included female sex and low educational attainment. Enabling factors included low income and the inability to go out independently. Needs factors with a statistically significant association included external functional disabilities, mild disabilities, depression, and suicidal ideation.

**Conclusions:**

To address unmet medical needs positively, rather than controlling for nonmodifiable predisposing factors, greater consideration should be given to modifiable enabling and need factors. Therefore, improvements in economic conditions and mental healthcare should be prioritized. Further, enhancing healthcare accessibility tailored to the needs of people with disabilities, and implementing community-based public health and policy efforts to meet specific requirements are essential.

## 1 Introduction

As of 2024, approximately 2.64 million people in South Korea have been registered as having disabilities, accounting for 5.1% of the total population (1). This number has increased substantially from 240,000 in 1990, largely driven by factors such as population aging and changes in disability registration systems (2, 3).

Advancements in medical technology have extended the life expectancy of people with disabilities (PWD) and their proportion in the population is expected to continue to increase (4, 5). Therefore, the likelihood of encountering secondary disabilities and health complications will also rise (6–8). However, societal efforts to understand and address health challenges faced by PWD remain insufficient. Health policies often fail to adequately reflect the diverse needs and characteristics of target populations, resulting in limited support (9).

In particular, PWD report low accessibility to health services despite a greater need for health management (10, 11). They also experience more complex and time-consuming processes in accessing care and their need for health services tends to increase throughout their lives, further increasing the possibility of experiencing unmet healthcare needs (11–13).

High rates of unmet medical needs among PWD pose a critical threat to their right to healthcare, quality of life and, in severe cases, even survival (14, 15). Unmet medical needs are shaped by both health status and barriers to healthcare access; therefore, Andersen's behavioral model provides a useful framework for examining the determinants of unmet healthcare needs in this population. The model is one of the most widely used frameworks for understanding healthcare utilization and access to care. It proposes that healthcare use is determined by three domains: predisposing characteristics, enabling resources, and need factors. Predisposing characteristics refer to demographic and social factors that influence health-related behaviors, enabling resources refer to the means that facilitate or hinder access to healthcare services, and need factors represent perceived or evaluated health conditions requiring medical attention (16–18). We aimed to explore the factors associated with unmet healthcare needs among PWD by applying Andersen's behavioral model (19).

Previous studies on unmet healthcare needs have focused on equity, particularly disparities between people with and without disabilities (13, 20, 21). However, few recent studies have analyzed the determinants of unmet healthcare needs, specifically among PWD in Korea. Given the ongoing national discourse on economic hardship, health vulnerability, and gaps in medical services for people with disabilities (20, 22), this study aimed to identify the multifaceted factors contributing to their unmet needs by integrating predisposing, enabling, and need-based variables. Ultimately, the findings will be used to provide evidence-based insights for policy planning and implementation in healthcare for people with disabilities.

## 2 Materials and methods

### 2.1 Study design

**Figure 1:**
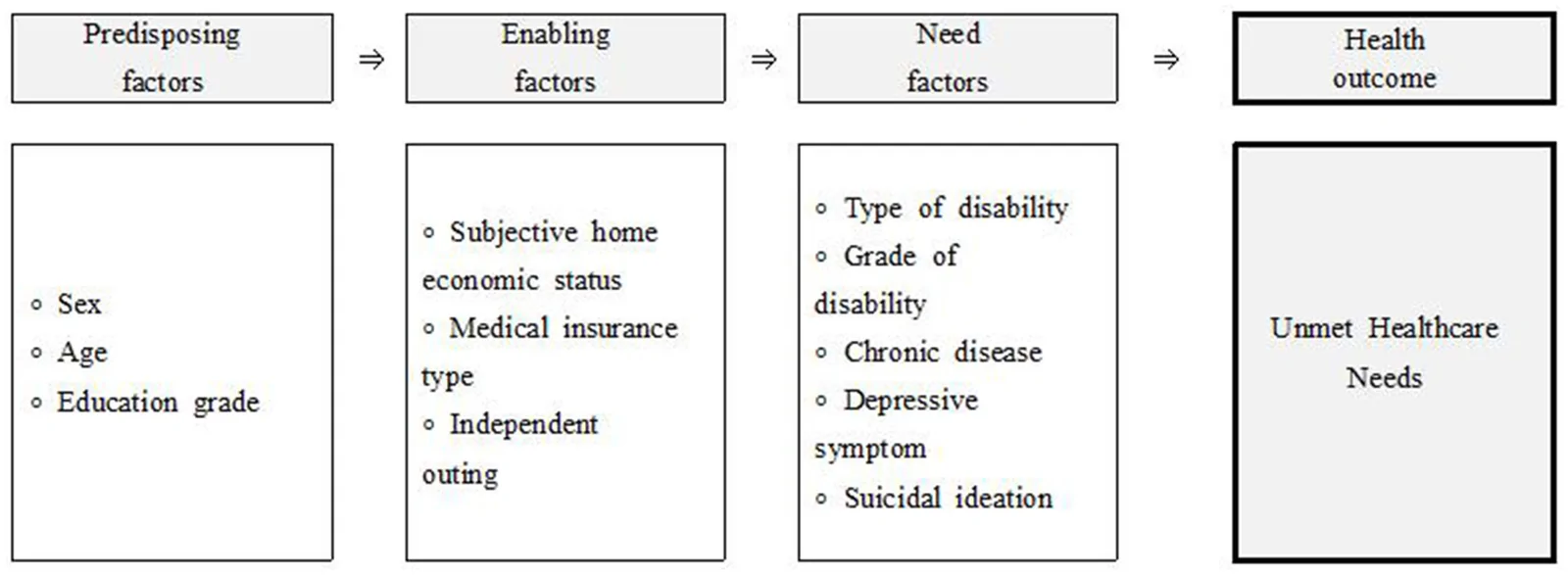
Study framework.

This study aimed to identify the factors associated with unmet medical needs among PWD in Korea based on Andersen's behavioral model (19). The study framework is outlined in Figure 1.

### 2.2 Study population and data

This study used raw data from the 2023 Survey on the Status of Persons with Disabilities conducted by the Korea Ministry of Health and Welfare and Institute for Health and Social Affairs (23). The survey was conducted between September and November 2023 and targeted individuals with disabilities registered in general residential facilities across 17 metropolitan cities and provinces nationwide as of May 2023. In total, 11,480 registered PWD were surveyed and 8,000 responded. Among them, 7,581 individuals aged ≥20 years were selected for analysis.

### 2.3 Variables

#### 2.3.1 Dependent variable

The experience of unmet medical needs was assessed based on the survey question: “In the past year (September 2022 to August 2023), have you ever wanted to visit a medical institution but could not?” PWD who answered “yes” were classified as having experienced unmet medical needs. This measure reflects self-reported unmet medical needs, operationally defined in the Survey on Persons with Disabilities.

#### 2.3.2 Independent variables

##### 2.3.2.1 Predisposing factors

Predisposing factors included demographic and sociostructural variables existing outside the will of an individual, such as sex, age, occupation, and education. In this study, sex and age were included as demographic variables. Education level was included as a sociostructural variable and was categorized into the following five groups: no schooling, elementary school graduate, middle school graduate, high school graduate, and college or higher.

##### 2.3.2.2 Enabling factors

According to Andersen's behavioral model, enabling factors include resources and conditions that facilitate or impede healthcare utilization. For people with disabilities, mobility and the ability to travel independently are important enabling resources that directly affect access to healthcare services (16, 17). Enabling factors were those that facilitated access to medical services by fulfilling healthcare needs, such as income and medical insurance coverage. In this study, the enabling factors were the subjective economic status, health insurance coverage, and whether the individual was able to leave the house independently.

##### 2.3.2.3 Need factors

Andersen's model conceptualizes need factors as health conditions that generate demand for healthcare services. Previous applications of Andersen's framework among vulnerable populations have similarly categorized mental health conditions as need factors (16, 19). Need factors referred to the relative necessity of medical services based on the illness. In this study, need factors included the disability type (external physical disabilities, internal organ disabilities, or mental disabilities), severity of disability (severe or mild), and presence of chronic disease, sadness or despair (depression), or suicidal ideation.

### 2.4 Data analysis

All statistical analysis was performed using IBM SPSS Statistics for Windows version 26.0 (IBM Corp., Armonk, NY, USA), with the significance level set at *p* < 0.05. The general and disability-related characteristics of participants were analyzed using frequencies, percentages, means, and standard deviations. Associations between predisposing, enabling, and need factors and unmet medical needs were examined using the chi-square test. A multiple logistic regression analysis was conducted, with experience of unmet medical needs as the dependent variable and predisposing, enabling, and need factors as independent variables. All independent variables were entered simultaneously into the multivariable logistic regression model using the enter method. The analyses were conducted using the original sample of 7,581 participants. Although the Survey on Persons with Disabilities provides sampling weights, the public-use dataset does not include variables for survey stratification and clustering. Therefore, complex survey analyses accounting for the full sampling design could not be performed. The primary objective of this study was to identify factors associated with unmet medical needs rather than to estimate nationally representative prevalence; therefore, multivariable logistic regression was performed using the original sample.

## 3 Results

### 3.1 General characteristics of study participants

The sociodemographic characteristics of the participants are shown in Table 1. Significant differences were found across all variables, which included: sex, age, highest education level, subjective economic status, type of medical coverage, ability to go out independently, type and severity of disability, presence of chronic disease, depression, and suicidal ideation.

**Table 1:** General characteristics of the study population: people with disabilities with and without unmet medical needs (*n* = 7,581) (unit: person, %).

Characteristic		PWD with unmet medical needs (*n* = 1,301)		PWD without unmet medical needs (*n* = 6,280)		*p*
Sex	Male	689	(53.0)	3,885	(61.9)	35.701^**^
	Female	612	(47.0)	2,395	(38.1)	
Age (years)	20~39	95	(7.3)	580	(9.2)	37.542^**^
	40~59	320	(24.6)	1,667	(26.5)	
	60~79	585	(45.0)	3,013	(48.0)	
	≥80	301	(23.1)	1,020	(16.2)	
	Mean±SD	65.61 ± 16.187		63.18 ± 16.110		24.424^**^
Education level	≤ Elementary	161	(12.5)	385	(6.2)	111.049^**^
	Middle school	235	(18.2)	956	(15.4)	
	High school	367	(28.4)	2,083	(33.5)	
	Vocational education program for students with disabilities	356	(27.6)	1,478	(23.8)	
	≥College	172	(13.3)	1,314	(21.1)	
Subjective home economic status	High level	91	(7.0)	911	(14.5)	119.376^**^
	Middle level	482	(37.0)	2,816	(44.8)	
	Low level	728	(56.0)	2,553	(40.7)	
Medical insurance type	NHI^1)^	952	(79.4)	5,067	(80.7)	35.613^**^
	Medical aid	346	(20.6)	1,212	(19.3)	
Independent outing	Yes	882	(67.8)	5,214	(83.0)	158.736^**^
	No	419	(32.2)	1,066	(17.0)	
Type of disability	External physical disabilities^2)^	968	(74.4)	4,152	(66.1)	36.045^**^
	Internal organ disabilities^3)^	177	(13.6)	1,234	(19.6)	
	Mental disabilities^4)^	156	(12.0)	894	(14.2)	
Grade of disability	Severe	675	(51.9)	2,905	(46.3)	13.683^*^
	Mild	626	(48.1)	3,375	(53.7)	
Chronic disease	Yes	1,159	(89.1)	5,330	(84.9)	15.513^*^
	No	142	(10.9)	950	(15.1)	
Depressive symptoms	Yes	294	(22.6)	748	(11.9)	10.827^*^
	No	1,007	(77.4)	5,532	(88.1)	
Suicidal ideation	No	1,088	(83.6)	5,575	(92.0)	87.233^**^
	Yes	213	(16.4)	505	(8.0)	

Percentages were calculated using valid responses only; therefore, missing values were excluded from the denominator for each variable. Sample sizes may vary across variables because of missing data. NHI, National Health Insurance; PWD, people with disabilities. ^1)^National health insurance (employees', local). ^2)^Physical disability, Disability of brain lesion, Visual impairment, Hearing impairment, Speech disability, Facial dysfunction. ^3)^Kidney dysfunction, Cardiac dysfunction, Respiratory dysfunction, Hepatic dysfunction, Intestinal fistula/urinary fistula, Epilepsy. ^4)^Intellectual disability, Autism disorder, Mental disorder. ^*^p < 0.05, ^**^p < 0.001.

Among those who experienced unmet medical needs, the male-to-female ratio was approximately 5.3:4.7. In contrast, the ratio was approximately 6.2:3.8 among those without unmet needs. The average age of people with unmet medical needs was 65.6 years, which was approximately 2 years older than those without. Among participants with unmet medical needs, 28.4% were high school graduates, 27.6% had completed a vocational education program for students with disabilities, 18.2% had graduated from middle school, 13.3% had graduated from college or higher, and 12.5% had completed elementary school or less. The distribution of medical coverage types was similar in both groups, with an approximate ratio of 8:2 of National Health Insurance to medical aid. However, the proportion of medical aid recipients was slightly higher among patients with unmet medical needs. Additionally, 56.0% of those with unmet medical needs rated their subjective home economic status as low, 37.0% as medium, and 7.0% as high. Among those without unmet needs, the distribution was 44.8% middle, 40.7% low, and 14.5% high.

Among PWD with unmet medical needs, 32.2% reported no ability (“independent outing”), compared with 17.0% of PWD without unmet medical needs (*p* < 0.001).

The most common types of disabilities in both groups were external physical disabilities, followed by internal organ and mental disabilities. In addition, 51.9% of those with unmet medical needs had severe disabilities, compared with only 46.3% of those without unmet needs.

The incidence of chronic diseases was high in both groups; however, both groups had higher proportions of participants with no depressive symptoms. Suicidal ideation was low in both groups, although it was reported in 16.4% of those with unmet medical needs compared to 8.0% of those without.

### 3.2 Experience of unmet needs of people with disabilities

Approximately 17.2% of people with disabilities reported that they wanted to visit medical institutions, but were unable to do so. The primary reason, accounting for 37.0%, was “difficulty in transportation to medical facilities,” followed by “economic reasons” at 26.0%. Other reasons included “lack of time” (10.0%), “no one to accompany when visiting medical institutions” (8.7%), “mild symptoms” (5.8%), and “communication difficulties” (5.0%) (Table 2).

**Table 2:** Participation rate of and cause of unmet medical needs of people with disabilities (unit: person, %).

Variable	PWD with unmet medical needs (*n* = 1,301)		PWD without unmet medical needs (*n* = 6,280)	
Participation rates of people with unmet medical needs	1,301	(17.2)	6,280	(82.8)
Cause of unmet medical needs				
Poor transportation service	481	(37.0)		
Economic problems	338	(26.0)		
Not enough time to visit the hospital	130	(10.0)		
No one to go to the hospital with	113	(8.7)		
Mild symptoms	76	(5.8)		
Communication problems	65	(5.0)		
Inadequate facilities for people with disabilities in the medical center	38	(2.9)		
Difficult to reserve	16	(1.2)		
Long wait at the medical center	14	(1.1)		
Difficulty in choosing a medical provider	11	(0.8)		
Lack of knowledge about health screening for disabled people among medical officials	8	(0.6)		
Other	11	(0.8)		

PWD, people with disabilities.

### 3.3 Analysis of factors associated with unmet medical needs among people with disabilities

Multiple logistic regression analysis was conducted to identify the factors associated with unmet medical needs among PWD (Table 3). The results showed that sex, highest educational level, subjective economic status, ability to go out independently, type and severity of disability, depression, and suicidal ideation had significant effects.

**Table 3:** Factors associated with unmet medical needs of people with disabilities in the multiple logistic regression analysis.

	Variable		OR	95% CI
Predisposing factors	Sex	Male	1	
		Female^*^	1.166	(1.022–1.330)
	Age group (years)	≥80	1	
		60~79	0.881	(0.740–1.049)
		40~59	1.116	(0.890–1.400)
		20~39	1.270	(0.904–1.785)
	Education level	≥College	1	
		High school^*^	1.456	(1.158–1.830)
		Vocational education program for students with disabilities	1.184	(0.967–1.449)
		Middle school^*^	1.649	(1.306–2.080)
		≤ Elementary^*^	2.123	(1.595–2.826)
Enabling factors	Subjective home economic status	High^1)^	1	
		Middle^*^	1.603	(1.255–2.049)
		Low^*^	2.394	(1.861–3.079)
	Medical insurance type	NHI	1	
		Medical aid	1.054	(0.890–1.248)
	Independent outing	Yes	1	
		No^*^	1.886	(1.624–2.191)
Need factors	Type of disability	Mental disabilities^2)^	1	
		Internal organs disabilities^3)^	1.173	(0.900–1.531)
		External physical disabilities^4)^	1.849	(1.464–2.336)
	Grade of disability	Severe	1	
		Mild^*^	1.175	(1.021–1.351)
	Chronic disease	Yes	1	
		No	1.149	(0.930–1.420)
	Depressive symptom	No	1	
		Yes^*^	1.563	(1.292–1.890)
	Suicidal ideation	No	1	
		Yes^*^	1.416	(1.139–1.759)

^1)^National health insurance (employees', local). ^2)^Intellectual disability, Autism spectrum disorder, Mental disorder. ^3)^Kidney dysfunction, Cardiac dysfunction, Respiratory dysfunction, Hepatic dysfunction, Intestinal fistula/urinary fistula, Epilepsy. ^4)^Physical disability, Disability of brain lesion, Visual impairment, Hearing impairment, Speech disability, Facial dysfunction. ^*^p < 0.05. CI, confidence interval; NHI, National Health Insurance; OR, odds ratio.

Females were 1.2 times more likely than males to experience unmet medical needs [95% confidence interval (CI), 1.022–1.330].

Compared with PWD who had graduated from college or higher, those with an elementary school education or less had higher odds of reporting unmet medical needs [odds ration (OR) = 2.12, 95% CI, 1.595–2.826). People with a middle school diploma were 1.6 times more likely (95% CI, 1.306–2.088), and those with an elementary school diploma were 1.65 times more likely (95% CI, 1.595–2.826) to experience unmet medical needs.

Compared with the high economic status group, the middle group was 1.6 times (95% CI, 1.255–2.049) and the low group was 2.4 times (95% CI, 1.861–3.079) more likely to have experienced unmet medical needs.

Those unable to go out independently were 1.9 times more likely to have unmet medical needs than those who were able to go out independently (95% CI, 1.624–2.191).

People with external functional disabilities were 1.8 times more likely to experience unmet medical needs than those with mental disabilities (95% CI 1.464–2.336).

People with mild disabilities were 1.2 times more likely to experience unmet medical needs than those with severe disabilities (95% CI, 1.021–1.351).

People experiencing depression were 1.6 times more likely to have unmet medical needs than those who were not (95% CI 1.292–1.890).

Finally, people with suicidal ideation were 1.4 times more likely to have unmet medical needs than those without (95% CI, 1.139–1.759).

## 4 Discussion

This study aimed to examine the extent of unmet medical needs among PWD in Korea and toidentify predisposing, enabling, and need factors associated with unmet medical needs based on Andersen's behavioral model.

The results showed that the prevalence of unmet medical needs among PWD in Korea was 17.2%. A previous study reported a similar figure of 17.7% (24). Using data from the National Population Health Survey, McColl et al. (2010) analyzed unmet medical needs among people aged 20–64 years with and without disabilities, and found rates of 17.5% and 5.2%, respectively (12). Mitra et al. (2011) used data from the 2007 Massachusetts Survey and reported that 69.6% of working adults aged 18–59 with disabilities experienced unmet medical needs at least once, with higher rates among those with activity limitations (25). A study using Canadian Community Health Survey data found that individuals with disabilities were over four times more likely to report unmet healthcare needs than those without (26). Although direct comparisons are limited, these findings indicate a high prevalence of unmet medical needs among individuals with disabilities.

The leading reason people with disabilities were unable to visit medical institutions, despite wanting to, was “inconvenient transportation,” accounting for 37.0%, followed by “economic reasons” at 26.0%. Other reasons included “lack of time” (10.0%), “no one to accompany when visiting medical institutions” (8.7%), “mild symptoms” (5.8%), and “difficulty using medical facilities” (5.0%). These results highlight the urgent need to develop effective measures to reduce the unmet medical needs of PWD.

The higher probability of experiencing unmet medical needs in women was consistent with previous findings that women with disabilities were 7.2 times (95% CI 2.7–19.4) more likely to have unmet needs related to treatment or medication costs compared with non-disabled men (10).

Our study supported previous research indicating that people with disabilities who had lower economic status experienced greater unmet medical needs (12, 27). This relationship underscores how economic constraints critically limit healthcare accessibility for people with disabilities. For example, Shin (2013) noted that, in Korea, this issue is linked to limitations in the coverage of the National Health Insurance (20).

Our data showed that people with external physical disabilities also had a higher likelihood of unmet medical needs than those with mental disabilities. Similarly, Choi et al. (2021) found that people with external physical disabilities have higher rates of unmet medical needs than those with other disability types (28), likely owing to substantial physical and environmental access barriers.

Our study identified the ability to go out independently as one of the strongest factors associated with unmet medical needs. In fact, “difficulty in transportation to medical institutions” was the primary reason for unmet needs, indicating that physical accessibility constraints pose a serious barrier to healthcare utilization among people with disabilities. Rotarou and Sakellariou (2019) reported similar findings in Greece, where limited transportation infrastructure, often because of austerity measures, prevented people with disabilities from visiting hospitals [10]. Kwon (2018) also pointed out disparities in regional healthcare access and a lack of support systems for transportation-vulnerable groups among people with disabilities in Korea (13). These findings suggest that improving transportation accessibility may help reduce barriers to healthcare among PWD. However, the present study did not directly evaluate the effectiveness of transportation support policies, and further research is warranted.

Our results demonstrated that individuals with depression and suicidal ideation were significantly more likely to experience unmet medical needs. This suggests that individuals with mental distress are concurrently marginalized in accessing medical services, reflecting the limitations of separate healthcare systems for psychological and physical care. Okoro et al. (2014) reported that comorbidities increase the complexity of healthcare access (29), and Hamilton et al. (2022) highlighted barriers to healthcare for people with disabilities who have mental health issues (30).

This study had several limitations. First, the Survey on People with Disabilities was a cross-sectional study based on self-reporting. Responses to questions about unmet medical needs might have been influenced by survey design effects, question phrasing, response type, categorization, and the placement of questions within the survey. Participant selection, response, recall, and other biases might have affected the results. Second, as survey respondents were community-dwelling PWD, the findings may have limited generalizability to all people with disabilities. Third, because this study used a cross-sectional design, causal relationships between the identified factors and unmet medical needs could not be established. Therefore, the findings should be interpreted as associations rather than evidence of causality. Fourth, the public-use dataset did not provide information on survey stratification and clustering. Consequently, complex survey analyses accounting for the full sampling design could not be conducted. Although the study focused on identifying factors associated with unmet medical needs rather than estimating population prevalence, caution is warranted when generalizing the findings to the entire population of PWD in Korea. Finally, the Andersen model variables in this study did not include many potentially important factors associated with unmet medical needs. Therefore, relevant factors may have been omitted. Future studies should incorporate additional enabling and need-related variables, including transportation availability, caregiver support, disability duration, healthcare utilization, and functional status, to better explain unmet medical needs and reduce the possibility of confounding.

This study confirmed that unmet medical needs among people with disabilities are not simply individual issues, but the result of complex socio-structural factors. Women, individuals with lower incomes or less education, and those with limited ability to go out have significantly lower healthcare accessibility, and these factors pose crucial public health concerns. Accordingly, policy recommendations include expanding medical cost support for individuals with disabilities who have low income, and enhancing transportation support services and home-visit medical services to ensure mobility rights. In addition, the observed differences by disability type suggest that healthcare accessibility may vary according to disability characteristics. These findings may inform the development of disability-specific strategies to improve healthcare accessibility, although the effectiveness of such approaches should be evaluated in future studies. Future research should further examine disparities by geographic region, disability type, and temporal trends to better understand unmet medical needs among people with disabilities.

## 5 Conclusions

This study identified predisposing, enabling, and need factors associated with unmet medical needs among PWD in Korea. Despite the enactment of the Act on Health Rights and Medical Accessibility for Persons with Disabilities in 2017, our findings suggest that disparities in healthcare access remain, with differences observed according to disability characteristics. These findings highlight the need to consider the diverse characteristics and healthcare needs of PWD when developing strategies to improve healthcare accessibility. Community-based public health initiatives and disability-responsive healthcare services may help reduce unmet medical needs; however, the effectiveness of specific interventions should be evaluated in future studies. Overall, the present findings provide evidence that may support the development of more equitable and accessible healthcare policies for PWD in Korea.

## Data Availability

Publicly available datasets were analyzed in this study. The datasets analyzed for this study are derived from the 2023 Survey on Persons with Disabilities conducted by the Ministry of Health and Welfare and the Korea Institute for Health and Social Affairs. The data are available through the Microdata Integrated Service (MDIS) of Statistics Korea (https://mdis.kostat.go.kr), and access is subject to approval.
